# Metabolic fingerprints of fear memory consolidation during sleep

**DOI:** 10.1186/s13041-021-00733-6

**Published:** 2021-02-10

**Authors:** Iyo Koyanagi, Kazuhiro Sonomura, Toshie Naoi, Takaaki Ohnishi, Naoko Kaneko, Kazunobu Sawamoto, Taka-Aki Sato, Masanori Sakaguchi

**Affiliations:** 1 International Institute for Integrative Sleep Medicine (WPI-IIIS), University of Tsukuba, Tsukuba, Ibaraki Japan; 2 Doctoral Program in Neuroscience, Degree Programs in Comprehensive Human Sciences, Graduate School of Comprehensive Human Sciences, University of Tsukuba, Tsukuba, Ibaraki Japan; 3 Life Science Research Center, Technology Research Laboratory, Shimadzu Corporation, Kyoto, Japan; 4 Graduate School of Artificial Intelligence and Science, Rikkyo University, Tokyo, Japan; 5 Department of Developmental and Regenerative Neurobiology, Institute of Brain Science, Nagoya City University Graduate School of Medical Sciences, Nagoya, Aichi 467-8601 Japan; 6 Division of Neural Development and Regeneration, National Institute for Physiological Sciences, Okazaki, Aichi 444-8585 Japan; 7 R&D Center for Precision Medicine, University of Tsukuba, Tsukuba, Ibaraki Japan

**Keywords:** Fear memory, REM sleep, Non-REM sleep, Dentate gyrus, Hippocampus, Metabolomics, Purine metabolism

## Abstract

Metabolites underlying brain function and pathology are not as well understood as genes. Here, we applied a novel metabolomics approach to further understand the mechanisms of memory processing in sleep. As hippocampal dentate gyrus neurons are known to consolidate contextual fear memory, we analyzed real-time changes in metabolites in the dentate gyrus in different sleep–wake states in mice. Throughout the study, we consistently detected more than > 200 metabolites. Metabolite profiles changed dramactically upon sleep–wake state transitions, leading to a clear separation of phenotypes between wakefulness and sleep. By contrast, contextual fear memory consolidation induced less obvious metabolite phenotypes. However, changes in purine metabolites were observed upon both sleep–wake state transitions and contextual fear memory consolidation. Dietary supplementation of certain purine metabolites impaired correlations between conditioned fear responses before and after memory consolidation. These results point toward the importance of purine metabolism in fear memory processing during sleep.

## Introduction:

Sleep and memory tightly influence each other. Sleep deprivation generally impairs memory consolidation, whereas strong emotional experience often disturbs sleep [1]. These relationships may take aberrant courses in psychiatric disorders such as post-traumatic stress disorder, which often involves chronic sleep impairments [2]. Many aspects of traumatic memory can be studied using fear conditioning paradigms, in which animals learn to associate a conditioned stimulus with a fearful experience (e.g., foot shock) [3, 4]. The consolidation of contextual fear memories requires a protein synthesis-dependent process in the hippocampus [5]. Recently, the hippocampal dentate gyrus (DG) was shown to be necessary and sufficient for holding contextual fear memory trace (or *engram*) [6, 7]. Moreover, we recently found that the sparse activity of adult-born DG neurons during sleep is necessary for fear memory consolidation [8]. Thus, the DG is a key brain region for analyzing molecular mechanisms of associative memory processing during sleep.

In mice, sleep can be divided into rapid eye movement (REM) non-REM (NREM) states based on signatures of global neuronal oscillatory activity observed in the electro-encephalogram (EEG). These two sleep states are functionally correlated with different aspects of memory processing, including memory consolidation and extinction at the behavioral level, memory replay at the neuronal circuit level, and synaptic plasticity at the cellular and subcellular levels [8–19]. Sleep also contributes to the clearance of metabolites in the brain [20] via the so-called glymphatic system [21], which is functionally relevant to brain disorders such as Alzheimer’s disease [22]. Although metabolomic screening has separately revealed molecular signatures of sleep and memory processing (e.g., [22]), the metabolites associated with memory processing during sleep have not been identified. As recent advances in mass spectrometry have made metabolomic screening extremely sensitive and high-throughput [24], this provides an opportunity to examine metabolic dynamics during memory processing in sleep.

Here, we performed unbiased metabolomics screening in the DG to identify metabolites relevant to contextual fear memory processing during sleep in mice. By combining real-time sleep state judgments and high-throughput mass spectrometry, we found drastic changes in metabolites between wakefulness and sleep, which made it possible to accurately predict mouse sleep–wake states. Moreover, we found sleep state-specific purine metabolic changes before and after memory consolidation, and the dietary supplementation of purines altered correlations between conditioned fear responses across the memory consolidation period. These results reveal the metabolic fingerprints of fear memory processing during sleep.

## Results:

### Metabolomic changes during sleep

**Fig. 1:**
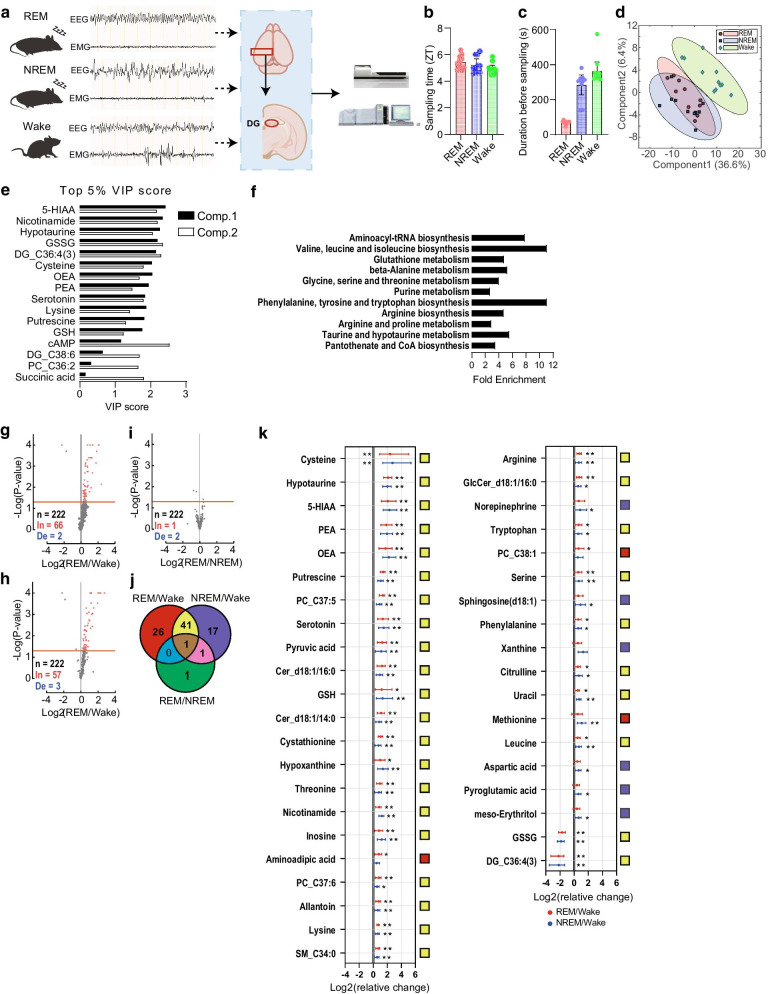
Metabolomic changes during sleep–wake states. **a** Real-time sleep state analysis, DG dissection, and metabolomic analysis using mass spectrometry. **b** Timing of tissue sampling (ZT = 0 indicates start of light cycle). **c** Average duration between detection of target sleepwake state and tissue sampling. **d** PLS-DA plot showing significant separation of the Wake group from the REM and NREM groups. Each point reflects one mouse, and ellipses represent 95% CIs. REM, *n* = 10 mice; NREM, *n* = 10 mice; Wake, *n* = 11 mice. **e** Top 5% of metabolites with the highest VIP scores. **f** Enrichment analysis of the top 5% of metabolites with the highest VIP scores listed in order of their statistical significance (i.e., smallest *p*-value at top). **g-i** Volcano plots of *p*-values of each metabolite from permutation analysis: REM vs. Wake (G), NREM vs. Wake (H), and REM vs. NREM (I). Red dashed line, *p* = 0.05; In, increase; De, decrease. **j** Venn diagram indicating numbers of significantly changed metabolites for each comparison (e.g., REM/Wake means significantly changed, either positively or negatively, in REM vs. Wake groups). **k** Fold change of each metabolite (*|fold change|> 1.5 and *p* < 0.05, **|fold change|> 1.5 and *p* < 0.01), sorted from the highest fold change for REM vs. Wake. Error bars, 95% CIs. *p < 0.05, **p < 0.01

We first examined changes in metabolites in the DG across sleep–wake states (Fig. 1a). Sleep–wake states were monitored by EEG in real time, after which DG tissue was obtained at zeitgeber time (ZT) = 5.2 ± 0.1 (mean ± standard error of the mean (SEM); Fig. 1b), chosen based on previous observations that DG neurons engage in fear memory consolidation during this circadian time period [8]. DG tissue was collected when mice remained in REM sleep, NREM sleep, or wakefulness (REM, NREM, and Wake groups, respectively) longer than pre-determined threshold time periods (Fig. 1c). The threshold period for REM sleep (1 min) was shorter than those for NREM sleep (5 min) and wakefulness (5 min) due to the brief length of single episodes of REM sleep (62.6 ± 5.5 s, mean ± SEM; Fig. 4c) [8]. Tissue was then processed for mass spectrometry, which consistently identified > 200 metabolites across samples (*n* > 10 mice per group; Additional file 1).

For metabolites with measurable concentrations across sleep–wake states, we performed principle component analysis (PCA), an unsupervised method of analyzing clustering, trends, and outliers [25–27]. Outliers falling outside the 95% confidence interval (CI; Additional file 4: Fig. S1A) were not detected by PCA (*n* = 31 mice). Next, we performed partial least squares discriminant analysis (PLS-DA), a supervised machine learning method, to identify differences in metabolites among sleep–wake states. PLS-DA is a commonly used metabolomics approach that is well suited for an uneven design matrix, in which the number of dependent variables (i.e., analyzed metabolites) is greater than the number of independent variables (i.e., number of mice) [23]. We found that the top three components separated REM and NREM states from the Wake state (Fig. 1d; Q2 value: 0.52; permutation test, REM vs. Wake, *p* < 0.05, NREM vs. Wake, *p* < 0.05). Additionally, PLS-DA identified metabolites driving the separation among sleep–wake states by ascribing variable importance of project (VIP) scores (Fig. 1e; generally, VIP scores > 1.0 are considered to contribute to observed differences).

Enrichment analyses [28] using the top 5% of metabolites with the highest VIP scores identified several metabolic pathways contributing to the separation among sleep–wake states, including tRNA, amino acids, and purine metabolism (Fig. 1f). Consistently, pairwise comparisons using permutation analysis showed changes in individual metabolites (*p* < 0.05, fold change >|1.5|, *n* = 86 metabolites with some overlap) mostly fitting to these categories (Fig. 1g–k, Additional file 4: Fig. S1B, C). Notably, we found a decrease in GSSG and increases in GSH and allantoin during sleep. Glutathione is composed of cysteine, glutamic acid, and glycine and exists in reduced (GSH) and oxidized (GSSG) states, the ratio of which is a measure of oxidative stress. Also, cysteine, which functions as an anti-oxidative agent similar to allantoin (a metabolite in the purine metabolism pathway) [29], increased during sleep. These findings suggest that the DG creates an antioxidative environment during sleep by producing these metabolites, consistent with the known enhanced antioxidant capacity in the brain during sleep [30, 31].

### Metabolomic changes during fear memory consolidation

**Fig. 2:**
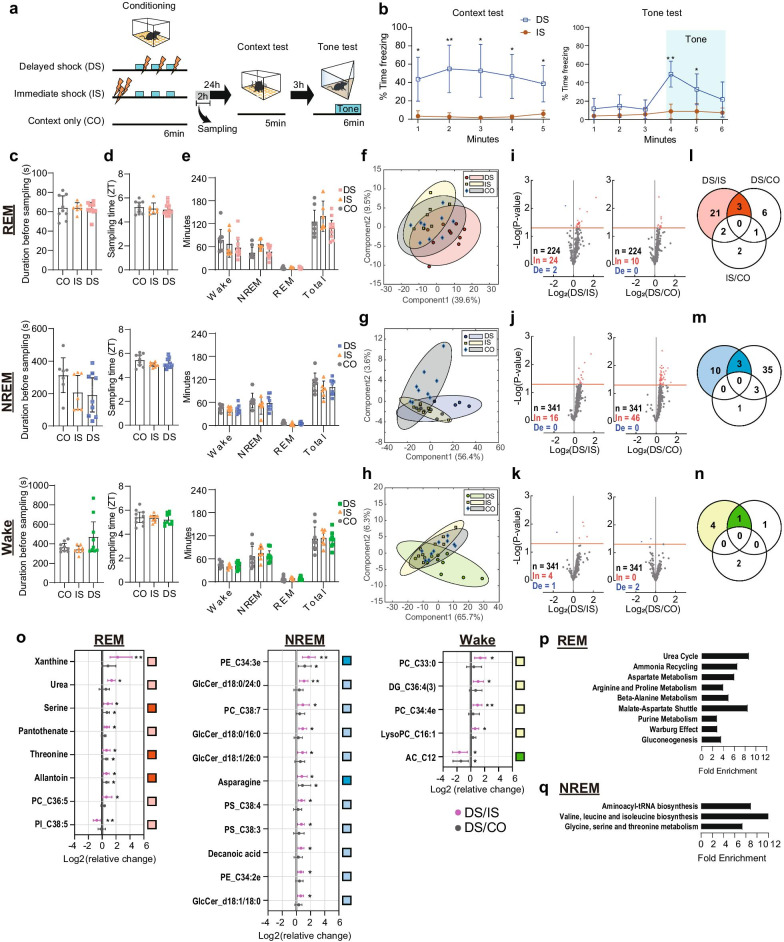
Metabolomic changes during fear memory consolidation. **a** Fear conditioning protocols and timing of tissue sampling for each group. **b** Freezing in the context (left) and tone (right) tests (DS, *n* = 8 mice; IS, *n* = 9 mice; two-tailed unpaired *t*-test, *p < 0.05, **p < 0.01; error bars, SEM). **c** Latency between detection of the target sleep–wake state and sampling. One-way ANOVA, *p* > 0.05, error bars, 95% CIs. **d** Sampling timing for REM (top), NREM (middle), and Wake (bottom) groups. ZT, zeitgeber time. ZT = 0 means the start of the light-cycle. One-way ANOVA, *p* > 0.05, error bars, 95% CIs. **e** Durations of each sleep–wake state between fear conditioning and sampling. Kruskal–Wallis test, *p* > 0.05, error bars, 95% CIs. PLS-DA plots for REM (**f**), NREM (**g**), and Wake (**h**) groups. Each point reflects one mouse, and ellipses represent 95% CIs. DS-REM, *n* = 12 mice; IS-REM, *n* = 7; CO-REM, *n* = 9; DS-NREM, *n* = 10; IS-NREM, *n* = 8; CO-NREM, *n* = 9; DS-Wake, *n* = 10; IS-Wake, *n* = 9; CO-Wake, *n* = 10. **i**–**k** Volcano plots of *p*-values of each metabolite from permutation analysis: DS vs. IS (left) and DS vs. CO (right) during REM sleep; DS vs. IS (up) and DS vs. CO (bottom) during NREM sleep; DS vs. IS (up) and DS vs. CO (bottom) during wakefulness. Red dashed line, *p* = 0.05. **l**–**n** Venn diagrams indicating numbers of significantly changed metabolites for each comparison (e.g., DS/IS means significantly changed, either positively or negatively, in DS vs. IS groups) during REM (left), NREM (middle), and Wake (right) groups. **o** Fold change of each metabolite (*|fold change|> 1.5 and *p* < 0.05, **|fold change|> 1.5 and *p* < 0.01), sorted from the highest fold change for DS vs. IS. Error bars, 95% CIs. Enrichment analysis for changed metabolites satisfying the criterion of $({\text{DS}}/\mathrm{IS})\bigcap (\overline{\mathrm{IS}\bigcap \mathrm{CO}})$) during REM (**p**) and NREM (**q**) sleep. No enriched pathways were found during wakefulness

Next, we examined metabolomic changes during fear memory consolidation in sleep. We employed a fear conditioning paradigm in which mice learn to associate a novel context (conditioned stimulus 1) and tone stimuli (conditioned stimulus 2) with aversive stimuli in the form of mild foot shocks (unconditioned stimulus) in a single session (delayed shock protocol (DS); Fig. 2a) as described previously [8]. In the DS protocol, mice explored a novel context for 3 min to establish a contextual memory, received three foot shocks co-terminating with tone stimuli, and remained in the context for another 30 s before being returned to their home cage. In subsequent test sessions in which mice were returned to the same context (i.e., context test) and then exposed to a different context in which the tone stimulus was replayed (i.e., tone test), mice showed the species-specific conditioned fear response of freezing (Fig. 2a, b) [32]. In sharp contrast, mice that underwent an immediate shock (IS) protocol in which they received foot shocks immediately upon entering the context spent much less time freezing during the context and tone tests (Fig. 2a, b), despite that both IS and DS mice received the same amount and intensity of conditioned and unconditioned stimuli. These results are consistent with our previous finding that our fear conditioning paradigm does not affect sleep architecture in the DS and IS groups [8]. As the low level of freezing exhibited by IS mice is considered to reflect a lack of association between conditioned and unconditioned stimuli [33–35], these mice served as a control group. In preparation for metabolomic screening, another group of control mice did not receive any foot shocks (i.e., context only (CO)).

After fear conditioning, DG tissue was removed for mass spectrometry when mice were in REM sleep (*n* > 9 mice), NREM sleep (*n* > 10), or wakefulness (*n* > 10) (Fig. 2a). The average amounts of time spent in each sleep–wake state before tissue sampling and the ZT were similar among DS, IS, and CO groups (Fig. 2c, d). The average duration between fear conditioning (i.e., first context exposure) and tissue sampling was also similar among groups (122 ± 6.9 min, mean ± SEM; Fig. 2e total), by which time critical molecular changes underlying fear memory consolidation were expected to occur in the DS mouse DG [e.g., 36, 37]. Furthermore, the durations of each sleep–wake state between conditioning and tissue sampling were also similar among groups (Fig. 2e). From this tissue, we identified > 200 metabolites in each group (Additional file 2).

**Fig. 3:**
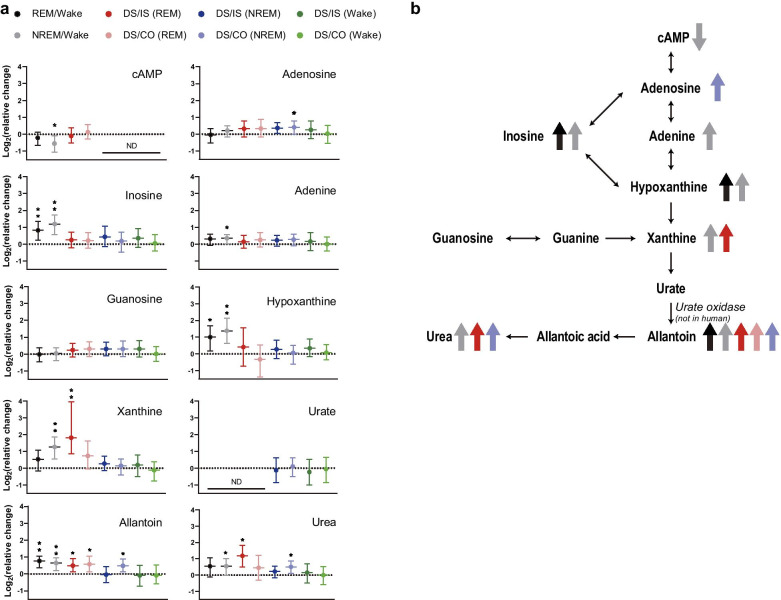
Changes in purine metabolism during sleep–wake state transitions and fear memory consolidation. **a** Fold changes of each metabolite in the purine metabolic pathway (e.g., REM/Wake indicates REM vs. Wake). Bars, mean; error bars, 95% CI; *p < 0.05, **p < 0.01 regardless of fold change; N.D., no data available. **b** Summary of results in **a**. Large arrows (color-coded as in **a**) indicate increases (pointing upward) or decreases (pointing downward) in the amount of each metabolite

To identify metabolites specific to the association between conditioned and unconditioned stimuli, we focused on differences among DS, IS, and CO groups within each sleep–wake state. PCA showed three outliers in the NREM group, which were omitted from further analysis (F Additional file 4: Fig. S2, arrows). Subsequent PLS-DA did not clearly separate the groups (Fig. 2f-h). However, permutation analysis (Fig. 2i-k) revealed changes in metabolites in a set of data satisfying the ($({\text{DS}}/\mathrm{IS})\bigcap (\overline{\mathrm{IS}/\mathrm{CO}})$) category (Fig. 2l-o, Additional file 4: Fig. S2E-G), which reflects the association between conditioned and unconditioned stimuli. Enrichment analysis of these metabolites showed fewer altered pathways (Fig. 2p, q) than those observed between sleep–wake states (Fig. 1f). However, a change in purine metabolism was a common trait between sleep–wake state transition and fear memory consolidation during sleep (Fig. 3a, b). These results suggest that purine metabolism in the DG is involved in fear memory processing during sleep.

### Effect of purine supplementation on sleep architecture and fear memory

As both sleep–wake states and fear memory consolidation during sleep were associated with changes in purine metabolites, we examined their effects on sleep architecture and fear learning and memory. As diet is the major source of purine metabolites, we chose metabolites that could be administered orally and transfer through the blood–brain barrier for immediate relevance to clinical application. We administered hypoxanthine [38, 39], xanthine [40, 41], allantoin [42, 43], and urea [44, 45] to mice for 1 week through their drinking water.

**Fig. 4:**
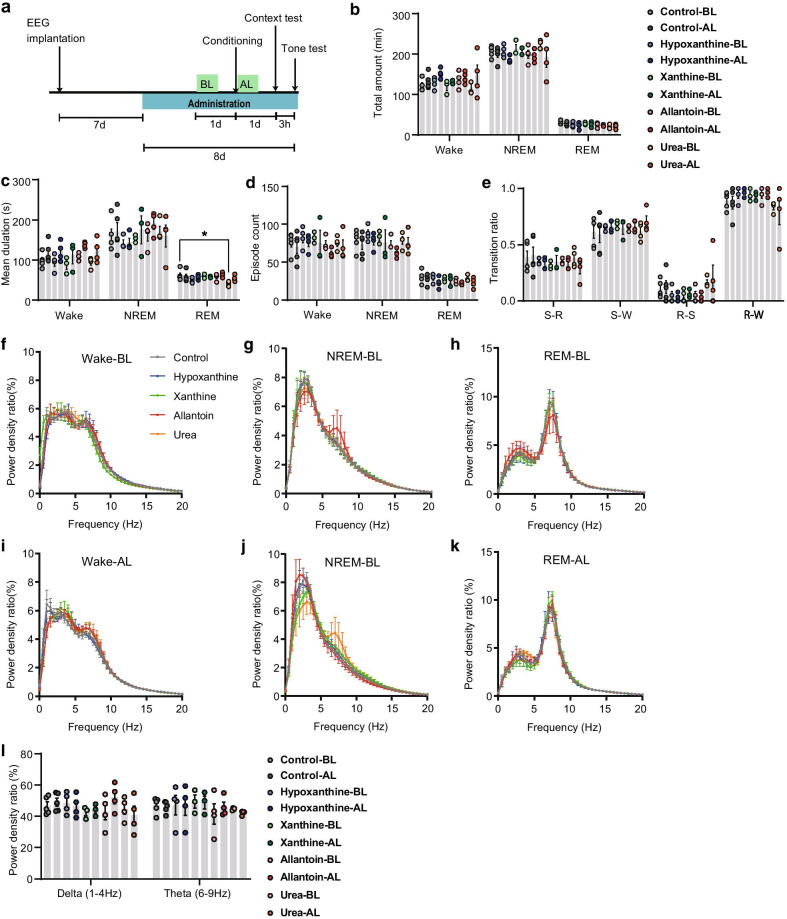
Effect of oral purine metabolite administration on sleep architecture. **a** Experimental schedule. BL, baseline EEG recording (ZT = 3–9); AL, EEG recording soon after learning (ZT = 3–9). **b**–**e** Sleep architecture during the recording periods: total amount (B), mean duration (C), and episode count (D) of each sleep–wake state and transition ratio between states (E)(control, *n* = 5 mice; hypoxanthine, *n* = 4; xanthine, *n* = 3; allantoin, *n* = 4; urea, *n* = 4; two-way ANOVA followed by Sidak’s multiple comparisons, control vs. urea, *p < 0.05, error bars, SEM). **(F-L)** Power analysis using Fast Fourier Transformation of EEG amplitude during BL (F–H) and AL (I-K) and their density (L; two-way ANOVA followed by Sidak’s multiple comparisons, *p* > 0.05, error bars, SEM)

We then examined sleep architecture 6 h after the DS protocol, which is during the memory consolidation period, and the corresponding circadian time period 24 h prior as a baseline (Fig. 4a; BL, baseline; AL, after learning). We observed no significant changes in the total amount, episode duration, or episode count of each sleep–wake state or their transition ratio during the baseline period among mice receiving normal drinking water or supplementation with hypoxanthine, xanthine, allantoin, or urea (Fig. 4b–e), except that mice receiving urea showed shorter REM sleep episodes. Power spectral analysis showed no changes among groups (Fig. 4f–l, Additional file 3).

**Fig. 5:**
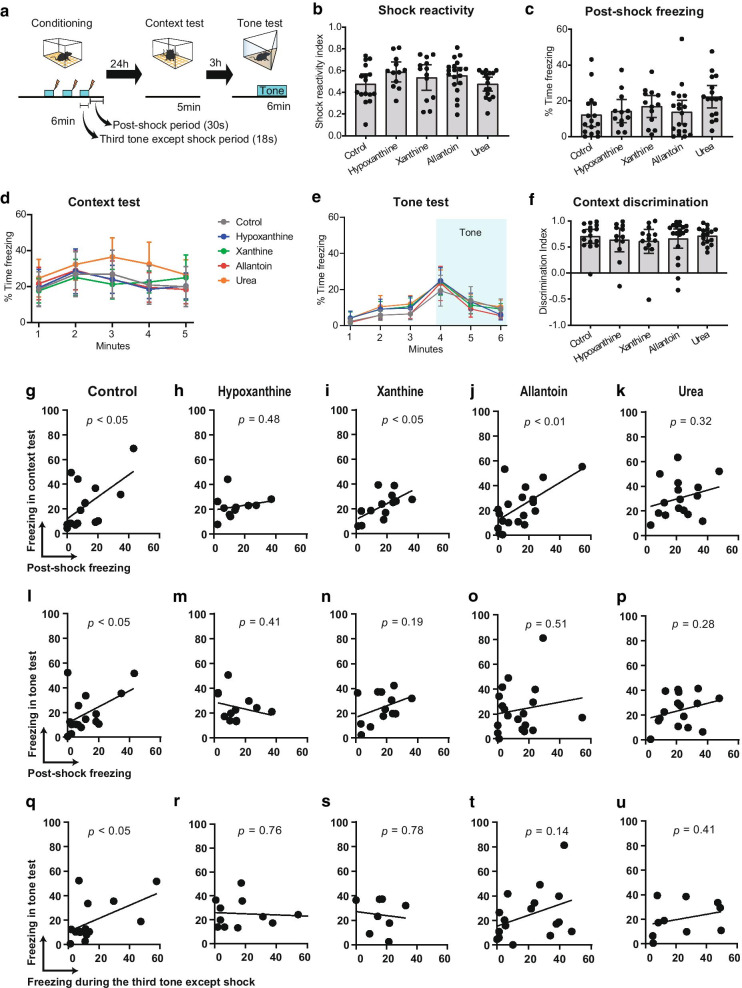
Effect of purine metabolite administration on fear memory. **a** Fear conditioning protocol (DS, same as in Figs. 2a and 4). Post-shock period denotes the 30-s period after the tone and shock toward the end of the session. **b** Shock reactivity during conditioning (one-way ANOVA, *p* > 0.05, error bars, 95% CIs). Freezing in post-shock period (**c**) and the context test (**d**), and the tone test (**e**; blue shade, timing of tone exposure) (one-way ANOVA, *p* > 0.05, error bars, 95% CIs). **f** Discrimination between the context used for the context test and that used for the tone test (Kruskal–Wallis test, *p* > 0.05, error bars, 95% CIs). **g**–**p** Correlations between freezing in post-shock period and freezing during the context test (**g**–**k** or tone test (**l**–**p**, during the first min of tone exposure). **q**–**u** Correlations between freezing in the third tone period (except the 2-s of shock period) and freezing during tone test. Two-tailed Pearson’s correlation coefficients, *p*-values indicated in each plot; trend lines, linear regression (**g**-**u**). Control, *n* = 16 mice; hypoxanthine, *n* = 12; xanthine, *n* = 13; allantoin, *n* = 19; urea, *n* = 16

The day after fear conditioning, we assessed fear memory in context (Fig. 5a, Additional file 4: Fig. S3A) and tone (Additional file 4: Fig. S3B) tests. There were no differences in shock reactivity (Fig. 5b) or movement (Additional file 4: Fig. S3C) during conditioning [46] among groups, indicating that all mice sensed the shocks and exhibited similar motor responses. Post-shock freezing, which represents a conditioned fear response [47, 48], was similar among groups, indicating normal fear memory expression (Fig. 5c). In addition, freezing levels in both the context (Fig. 5d) and tone (Fig. 5e) tests and discrimination between contexts (Fig. 5f) [49] were similar among groups.

Next, we analyzed correlations between conditioned responses before and after memory consolidation within individual mice. Consistent with a previous report [48], post-shock freezing was correlated with freezing in the context test in control, xanthine, and allantoin groups (Fig. 5g, i, j). However, there was no such correlation in hypoxanthine or urea groups (Fig. 5h, k). We also found a correlation between post-shock freezing and freezing in the tone test in the control group (Fig. 5l) but not in the purine-supplemented groups (Fig. 5m–p). As freezing was not induced by the tone during the post-shock period but was induced by the tone in the tone test, we also analyzed correlations between freezing in the third tone period during conditioning and the tone test. This correlation was significant in the control group (Fig. 5q) but not in the purine-supplemented groups (Fig. 5r–u). These results suggest that purine supplementation alters relationships between conditioned responses before and after fear memory consolidation.

## Discussion:

Our results reveal that DG metabolites dynamically change across sleep–wake states. Indeed, based on metabolomic phenotypes, it was possible to distinguish between mouse sleep and wakefulness states using a machine learning algorithm. By contrast, it was not possible to determine whether fear memory was established using the same methods, suggesting that the DG metabolome may be more strongly affected by sleep–wake state transitions than fear memory consolidation. These results are consistent with the fact that sleep provides a permissive environment for memory consolidation in the DG [8, 50]. Interestingly, changes in purine metabolism emerged as a common trait of both sleep–wake state transitions and fear memory consolidation, and dietary supplementation of some of these purines disrupted correlations between conditioned responses before and after memory consolidation.

Adenosine catabolism is the major source of purine metabolites, including hypoxanthine, xanthine, and uric acid (and its salt, urate), which are metabolized in this sequence. Previous studies show that prolonged wakefulness increases extracellular adenosine [51] and that adenosine regulates sleep, although its concentration in the basal forebrain is regulated by neuronal activity regardless of sleep–wake state [52]. Similarly, we did not find changes in adenosine level in the DG across sleep–wake states.

Lack of hypoxanthine–guanine phosphoribosyltransferase (HPRT) enzyme activity causes Lesch-Nyhan disease (LND) [53] by inducing systemic increases in the blood concentration of uric acid. Importantly, mice lacking HPRT expression do not recapitulate the neurological symptoms of LND or accumulate uric acid, making it difficult to study LND via loss of HPRT in rodents [54, 55]. This is because many non-human mammals metabolize uric acid into allantoin via urate oxidase. Thus, the lack of urate oxidase in humans may make them vulnerable to hyperuricemia [56], which may be associated with resilience to dementia [57]. In the present study, we did not detect a correlation between urate level and fear memory consolidation during sleep.

Both hyperuricemia and accumulation of hypoxanthine and xanthine may contribute to memory impairments. Indeed, intra-striatal hypoxanthine administration impairs fear memory learning, consolidation, and retrieval [58], and excessive hypoxanthine and xanthine induce oxidative stress [59], which inhibits Na^+^, K^+^-ATPase activity in the hippocampus and causes memory deficits [60, 61]. In contrast to our results, a previous study found that oral allantoin administration improves passive avoidance memory in mice [62]. We speculate that the reason for this discrepancy is that allantoin may induce different phenotypes depending on the memory paradigm [63]. Furthermore, the fine homeostatic regulation of purine concentration across sleep–wake transitions, as observed in this study, may be required for optimal memory consolidation during sleep. Further studies are needed to clarify this issue, as allantoin is a widely used dietary supplement for humans. In mice, allantoin is further metabolized into urea. A previous report shows that excessive dietary intake of the purine adenine results in high levels of urea in the brain, which impairs hippocampal synaptic plasticity by suppressing mTOR signaling [64].

Overall, this study reveals that DG metabolites change during memory consolidation in sleep. Future analyses of purine metabolism with higher spatial and temporal resolution will pave a way toward further understanding the significance of purine metabolism in memory consolidation during sleep.

## Methods:

### Animals

All experiments were performed in accordance with the Science Council of Japan’s Guidelines for Proper Conduct of Animal Experiments. Experimental protocols were approved by the Institutional Animal Care and Use Committee at the University of Tsukuba. C57BL/6 mice (Jackson Laboratory) were bred in our colony at the University of Tsukuba and maintained on a 12-h light/dark cycle (lights on 9 am-9 pm) with ad libitum access to food and water. All mice were group-housed with 2–5 mice/cage, and only male mice were used in the experiments.

### Surgical procedures and recording of EEG and EMG

Implantation of EEG/electro-myogram (EMG) sockets for recording was performed as previously described [65]. Briefly, the EEG/EMG recording electrode was composed of a six-pin header, six stainless steel wires (four 1.5-mm length and two 2.0-mm length), and four stainless steel screws (1.0-mm diameter). Mice were anesthetized with isoflurane (3–5%) during surgery. Using a carbide cutter (drill size: 0.8-mm diameter), four holes were made in the skull: two over the frontal cortical area (1.5 mm anterior to bregma, 1.5 mm lateral to midline) and two over the parietal area (3 mm posterior to bregma, 1.7 mm lateral to midline). Using a jeweler’s screwdriver, stainless steel EEG recording screws were placed into the holes. EMG recording wires were inserted into the trapezius (neck) muscle. After surgery, mice were handled for 2 min three times per day for 4 days and habituated to the recording chamber. The duration between electrode implantation surgery and subsequent behavioral testing was 7 days. Before behavioral testing, mice underwent baseline EEG/EMG recording in their home cage equipped with a data acquisition system (LabChart, AD Instruments, New Zealand; Vital recorder, KISSEI COMTEC, Japan). EEG/EMG signals were recorded during BL and AL periods for each mouse. EEG/EMG data were collected at a sampling rate of 128 Hz.

### Fear conditioning paradigm

The context used for fear conditioning consisted of a stainless steel chamber (31 × 24 × 21 cm; MED Associates) with a stainless steel shock grid floor (Additional file 4: Fig. S3A). The grid floor was composed of bars (3.2-mm diameter) spaced 7.9 mm apart that allowed the delivery of electric foot shocks. Under the grid floor was a stainless steel drop pan that was lightly cleaned with 75% ethanol, which also provided background odor. The front, top, and back of the chamber were made of clear acrylic, and the two sides were made of aluminum panels.

For the tone test, a different context was used that consisted of a white plastic floor covering the grid floor and a grey plastic triangular insert placed inside the chamber to create artificial left and right sides (Additional file 4: Fig. S3B). The front side consisted of a piece of cardboard with a blue and white rectangular pattern in the center. The context was cleaned with water instead of ethanol.

Mice in the DS group were placed in the conditioning context for 360 s, and three tones (30 s each, 2800 Hz, 85 dB) were played at 120, 210, and 300 s, with each tone co-terminating with a 2-s foot shock (0.75 s for Fig. 2). Post-shock freezing [47, 48] (Fig. 5c) was analyzed during the last 30 s of conditioning starting immediately after termination of the third tone, which was a tone- and shock-free period. Freezing during the third tone (Fig. 5l–p) was analyzed during the 28-s shock-free period of the third tone. We employed two additional control groups involving no context-shock association; mice in the IS group received three 2-s foot shocks at 2-s intervals immediately after being placed in the context, and mice in the CO group were exposed to the context for 360 s without receiving foot shocks. In these two control groups, tones were played at the same times as in the DS group. Mice were then returned to their home cage. Except for mice from which the DG was sampled, mice were later returned to the same context for 5 min without the delivery of foot shocks (i.e., context test). Three h later, mice were placed in a different context for 360 s, and the tone was played during the last 180 s (i.e., tone test). As we initially expected that allantoin administration would increase freezing levels [62], we weakened the intensity of the foot shocks (0.4 mA) in the oral administration experiments (Fig. 5) to clearly observe a potential enhancement of freezing. A discrimination index [3, 49] (Fig. 5f) was calculated as (freezing_contextA_ – freezing_contextB first 3 min_) /max(freezing_contextA_, freezing_contextB first 3 min_). A shock reactivity index during conditioning [66] (Fig. 5b) was calculated as (movement in the 2 s immediately before the first shock)/(movement in the 2 s of the first shock), and the nominator was also used to calculate relative movements (Additional file 4: Fig. S3C).

### Real-time sleep analysis and tissue preparation

Real-time EEG/EMG recordings to identify sleep–wake states were performed 1–3 h after conditioning. Mice were sacrificed when they exhibited 5 min of continued wakefulness, 5 min of continued NREM sleep, or 1 min of continued REM sleep. These thresholds of time spent in various sleep states were determined based on the average durations of states observed in mice. Mice were sacrificed by overdose of isoflurane within the sleep recording chamber by covering their nose with a 50-ml plastic tube containing paper soaked with liquid isoflurane and immediate decapitation. Their brains were removed and frozen at − 80 °C (< 3 min after completion of the necessary duration of each sleep state). All mice were briefly awake (< 5 s) during isoflurane inhalation before tissue sampling, which may have hindered the full discovery of small changes between sleep–wake states. Brains were sliced coronally (500 µm; from − 1.75 to − 2.25 mm relative to bregma) using a microtome. Slices were viewed under a microscope to dissect the DG in a frozen state.

### Sample preparation for brain metabolomic analysis

DG tissue was rapidly frozen in liquid nitrogen and stored at − 80 °C. Frozen tissue was homogenized using a manual homogenizer (BioMasherII, Nippi) in 150 µl ice-cold buffer (80% methanol containing 1% formic acid) with 6 µl of 0.1 mg/ml 2-isopropylmalic acid (Sigma-Aldrich), which was utilized as an internal standard. Samples with < 20 μg total protein were omitted from further analysis. After centrifugation at 16,000*g* for 30 min at 4 °C, 50 µl supernatant was dried in a centrifugal evaporator for GCMS analysis, and the remaining supernatant was used for LCMS analysis. Metabolites were quantified using GCMS (GCMS-QP2010 Ultra, Shimadzu) or LCMS (LCMS-8060, Shimadzu) as described previously [67]. Briefly, dried extract was subjected to methoximation followed by silylation, and 1 µl derivatized sample was injected into the GCMS system. For LCMS, polar metabolites were separated on a Shodex RSpak DE-213 column and measured with electrospray ionization in positive ion and multiple reaction monitoring mode. All ion transitions and collision energies for multiple reaction monitoring were optimized experimentally using authentic standards for each metabolite. Supernatant was directly injected into the LCMS system for lipid analysis and analyzed with hydrophilic interaction chromatography coupled with the electrospray ionization method [67].

### Drag administration

Allantoin (130 μmol/kg/day, Sigma-Aldrich, 05,670), xanthine (91 μmol/kg/day, Sigma-Aldrich, X7375), hypoxanthine (130 μmol/kg/day, Sigma-Aldrich, H9377), or urea (130 μmol/kg/day, Kanto Chemical Co. Inc., JAPAN, 43009-00) was dissolved in drinking water and administered to mice for 7 days before fear conditioning. Mice in the control group were given normal drinking water.

### Quantification and statistical analysis

Significant changes in metabolite levels were identified by PLS-DA using MetaboAnalyst (http://www.metaboanalyst.ca) [68, 69]. The quality of the PLS-DA models was assessed for R2, Q2, and accuracy based on a VIP score ≥ 1.0. Pathway analyses were performed using MetaboAnalyst. Pathways were considered affected if they were significantly enriched (*p* < 0.05) for all significantly altered metabolites. Three outliers were identified and excluded by PCA using MetaboAnalyst 4.0 as previously described [70]; these could have been due to sample degradation, instrumental error, changes in measurement conditions, or faulty measurements resulting from human error. To compare metabolite ratios among subgroups, bootstrapping to obtain 95% CIs and permutation analysis to obtain *p*-values were carried out by randomly replacing the measured value 10,000 times.

Statistical analysis was performed using GraphPad Prism version 8.4.0 for Windows (GraphPad Software, USA) or Igor Pro version 8.01B01 (Wave Metrics, USA). Type I error was set at 0.05. Shapiro–Wilk tests were performed to assess the normality of data. Brown-Forsythe tests were performed to assess homogeneity of variance. Other details of statistical analyses are described in the figure legends and below.

Figure 1b. REM, *n* = 10 mice; NREM, *n* = 10; Wake, *n* = 11, one-way ANOVA, *F*(2, 28) = 2.6, *p* = 0.095.

Figure 1k. See Additional file 1 for details of statistical analysis.

Figure 2b. DS (*n* = 8 mice) vs. IS (*n* = 9). Left (context test), two-way ANOVA, group × time, *F*(4, 60) = 2.67, *p* < 0.05; time: *F*(2.26, 33.9) = 1.17, *p* = 0.32; group, *F*(1, 15) = 24.5, *p* < 0.01; Sidak’s multiple comparisons tests, DS vs. IS, 1st min, *p* < 0.05, 2nd min, *p* < 0.01, 3rd to 5th min, *p* < 0.05. Right (tone test), two-way ANOVA, group × time, *F*(5, 75) = 15.0, *p* < 0.01; time: *F*(5, 75) = 24.7, *p* < 0.01; group: *F*(1, 15) = 8.85, *p* < 0.01; Sidak’s multiple comparisons tests, DS vs. IS, 1st to 3rd min, *p* > 0.5, 4th to 5th min, *p* < 0.01, 6th min, *p* = 0.16.

Figure 2c. Top (REM), DS, *n* = 11 mice; IS, *n* = 7; CO, *n* = 9; one-way ANOVA, *F*(2, 24) = 0.38, *p* = 0.69. Middle (NREM), DS, *n* = 10 mice; IS, *n* = 8; CO, *n* = 9; one-way ANOVA, *F*(2, 24) = 2.16, *p* = 0.14. Bottom (Wake), DS, *n* = 10 mice; IS, *n* = 9; CO, *n* = 10; one-way ANOVA, *F*(2, 26) = 0.30, *p* = 0.74.

Figure 2d

Top (REM), DS, *n* = 11 mice; IS, *n* = 7; CO, *n* = 9; one-way ANOVA, *F*(2, 24) = 0.046, *p* = 0.95.

Middle (NREM), DS, *n* = 9 mice; IS, *n* = 7; CO, *n* = 7; one-way ANOVA, *F*(2, 20) = 2.11, *p* = 0.15.

Bottom (Wake), DS, *n* = 9 mice; IS, *n* = 8; CO, *n* = 9; one-way ANOVA, *F*(2, 23) = 2.71, *p* = 0.087.

Figure 2e

Top (REM), DS, *n* = 10 mice; IS, *n* = 6; CO, *n* = 8; Kruscal–Wallies test, Wake, *p* = 0.19; NREM, *p* = 0.08; REM, *p* = 0.16; total, *p* = 0.28.

Middle (NREM), DS, *n* = 9 mice; IS, *n* = 6; CO, *n* = 7; Kruscal–Wallies test, Wake, *p* = 0.35; NREM, *p* = 0.56; REM, *p* = 0.19; total, *p* = 0.42.

Bottom (Wake), DS, *n* = 9 mice; IS, *n* = 7; CO, *n* = 8; Kruscal-Wallies test, Wake, *p* = 0.39; NREM, *p* = 0.49; REM, *p* = 0.53; total, *p* = 0.85. Figure 2O. See Additional file 2 for details of statistical analysis. Figure 3. See Additional file 1 and 2 for details of statistical analysis. Figure 4b-e, l. See Additional file 3 for details of statistical analysis. Figure 5b. Control, *n* = 16 mice; hypoxanthine, *n* = 12; xanthine, *n* = 13; allantoin, *n* = 19; urea, *n* = 16, one-way ANOVA, *F*(4, 71) = 1.36, *p* = 0.26. Figure 5c. Control, *n* = 16 mice; hypoxanthine, *n* = 12; xanthine, *n* = 13; allantoin, *n* = 19; urea, *n* = 16, Kruskal–Wallis test, *p* = 0.057. Figure 5d. Control, *n* = 16 mice; hypoxanthine, *n* = 12; xanthine, *n* = 13; allantoin, *n* = 19; urea, *n* = 16, two-way ANOVA, group × time, *F*(16, 284) = 0.88, *p* = 0.71; time, *F*(3.5, 246.6) = 5.9, *p* < 0.01; group, *F*(4, 71) = 0.90, *p* = 0.47. Figure 5e. Control, *n* = 16 mice; hypoxanthine, *n* = 12; xanthine, *n* = 13; allantoin, *n* = 19; urea, *n* = 16, two-way ANOVA, group × time, *F*(20, 355) = 0.75, *p* = 0.77; time, *F*(2.2, 156.6) = 65.5, *p* < 0.01; group, *F*(4, 71) = 0.84, *p* = 0.51.

Figure 5f. Control, *n* = 16 mice; hypoxanthine, *n* = 12; xanthine, *n* = 13; allantoin, *n* = 19; urea, *n* = 16, Kruskal–Wallis test, *p* = 0.88.

Figure 5g. *n* = 16 mice/group, two-tailed Pearson’s correlation, *r* = 0.58,* p* < 0.05.

Figure 5h. *n* = 12 mice/group, two-tailed Pearson’s correlation, *r* = 0.22,* p* = 0.48.

Figure 5i. *n* = 13 mice/group, two-tailed Pearson’s correlation, *r* = 0.62,* p* < 0.05.

Figure 5j. *n* = 19 mice/group, two-tailed Pearson’s correlation, *r* = 0.62,* p* < 0.01.

Figure 5k. *n* = 16 mice/group, two-tailed Pearson’s correlation, *r* = 0.27,* p* = 0.32.

Figure 5l. *n* = 16 mice/group, two-tailed Pearson’s correlation, *r* = 0.51,* p* < 0.05.

Figure 5m. *n* = 12 mice/group, two-tailed Pearson’s correlation, *r* = -0.26,* p* = 0.41.

Figure 5n. *n* = 13 mice/group, two-tailed Pearson’s correlation, *r* = 0.39,* p* = 0.19.

Figure 5o. *n* = 19 mice/group, two-tailed Pearson’s correlation, *r* = 0.16,* p* = 0.51. Figure 5p. *n* = 16 mice/group, two-tailed Pearson’s correlation, *r* = 0.28,* p* = 0.29.

Figure 5q, n = 14 mice/group, two tailed Peason’s correlation, *r* = 0.54, *p* < 0.05.

Figure 5r. *n* = 12 mice/group, two tailed Peason’s correlation, *r* = -0.10, *p* = 0.76.

Figure 5s. *n* = 8 mice/group, two tailed Peason’s correlation, *r* = -0.11, *p* = 0.78.

Figure 5t. *n* = 17 mice/group, two tailed Peason’s correlation, *r* = 0.38, *p* = 0.14.

Figure 5u. *n* = 10 mice/group, two tailed Peason’s correlation, *r* = 0.30, *p* = 0.41. Fig. S3C, Control, *n* = 16 mice; hypoxanthine, *n* = 12; xanthine, *n* = 13; allantoin, *n* = 19; urea, *n* = 16, Kruskal–Wallis test, *p* = 0.015; Dunn’s multiple comparisons test, no difference between control vs. hypothantine (*p* > 0.96), xanthine (*p* > 0.99), allantoin (*p* > 0.99), or urea (*p* > 0.085).

## Supplementary Information

**Additional file 1: Data S1.** Details of statistical analysis for Fig. 1

**Additional file 2: Data S2.** Details of statistical analysis for Fig. 2

**Additional file 3: Data S3.** Details of statistical analysis for Fig. 4

**Additional file 4: Fig. S1.** Metabolomic changes across sleep–wake states, related to Fig. 1. **(A)** PCA plots from each group of mice for each sleep–wake state. Each point reflects one mouse, and ellipses represent 95% CIs. **(B-C)** Fold change of each metabolite between REM vs. Wake groups; red, NREM vs. Wake; blue (B), REM vs. Wake; green (same as in B), REM vs. NREM (C). **Fig. S2.** PCA analysis found three outliers in the NREM group, related to Fig. 2 . **(A-D)** PCA plots from each group of mice in each sleep–wake state: REM sleep **(A)**, NREM sleep before (**B**) and after (**C**) eliminating outliers (two in the IS group, one in the DS group) that fell outside the 95% CIs ellipses, or wakefulness (**D**). Each point reflects one mouse. DS-REM group, *n* = 11 mice; IS-REM, *n* = 7; CO-REM, *n* = 9; DS-NREM, *n* = 10 (after eliminating one outlier); IS-NREM, *n* = 8 (after eliminating two outliers); CO-NREM, *n* = 9; DS-Wake, *n* = 10; IS-Wake, *n* = 9; CO-Wake, *n* = 10. Ellipses represent 95% CIs. **(E–G)** Fold change of each metabolite between DS vs. IS and DS vs CO in each sleep–wake state. **Fig. S3.** Front view of the fear conditioning context, related to Figs. 2, 4and 5. **(A-B)** Context A (**A**) for conditoining and the context test and Context B (**B**) for the tone test. (C) Relative movements during the pre-shock period. Control, *n* = 16 mice; allantoin, *n* = 19; xanthine, *n* = 13; hypoxanthine, *n* = 12; urea, *n* = 16. Kruskal–Wallis test, *p* < 0.05, Dunn’s multiple comparison tests, *p* > 0.05 for all comparisons, error bars, 95% CIs.

## Data Availability

All unique/stable reagents generated in this study are available from the corresponding author with a completed materials transfer agreement. Data underlying the results described in this manuscript are available at Mendeley Data: 10.17632/f6pbcvvbxb.1.

## Abbreviations

CO: Context only
DG: Dentate gyrus
DS: Delayed shock
EEG: Electro-encephalogram
EMG: Electro-myogram
GSH: Reduced glutathione
GSSG: Oxidized glutathione
HPRT: Hypoxanthine–guanine phosphoribosyltransferase
IS: Immediate shock
LND: Lesch-Nyhan disease
NREM: Non-rapid eye movement
PCA: Principal component analysis
PLS-DA: Partial least squares discriminant analysis
REM: Rapid eye movement
VIP: Variable importance of project
ZT: Zeitgeber time
