# Supplementary figures and images for: Metabolic fingerprints of fear memory consolidation during sleep
Source: Mol Brain. 2021 Feb 10;14:30. doi: 10.1186/s13041-021-00733-6 (PMC7874630; doi:10.1186/s13041-021-00733-6)

# Supplementary Figure 1

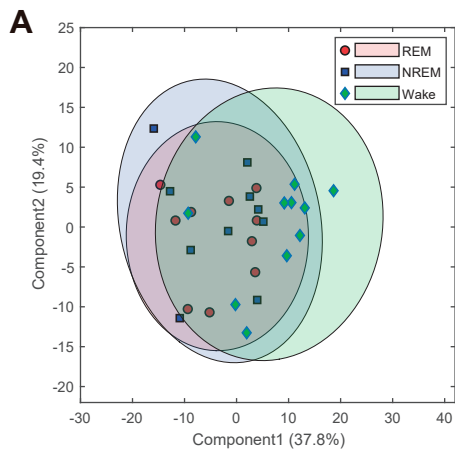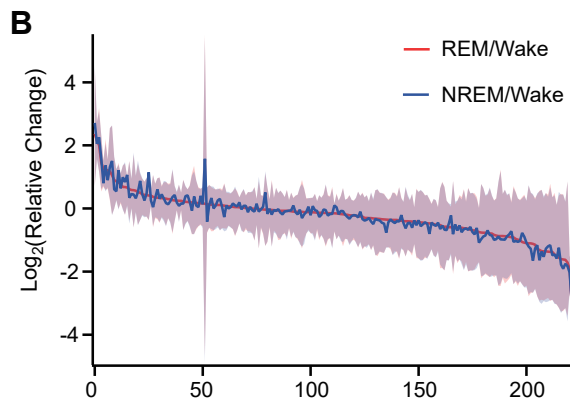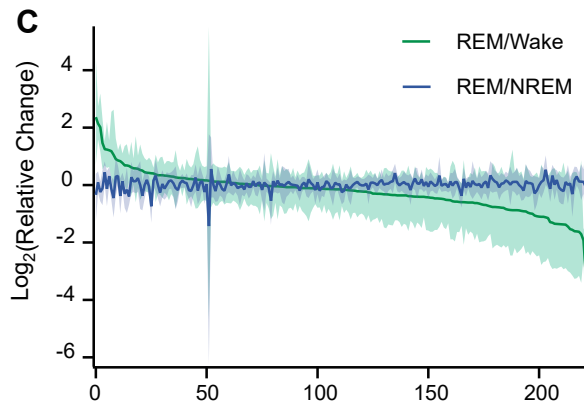

Supplementary Figure 2

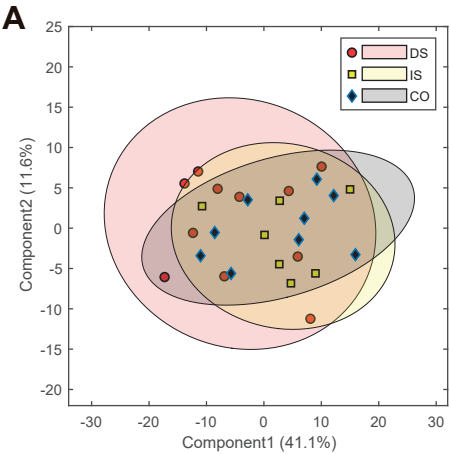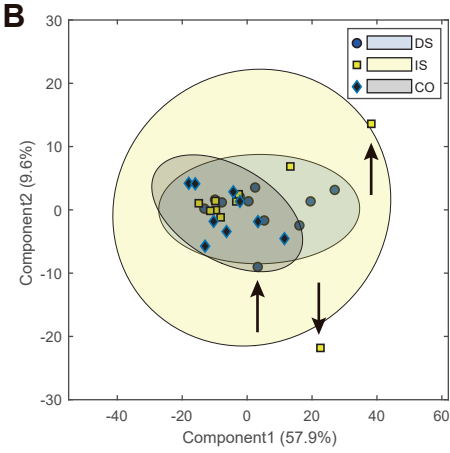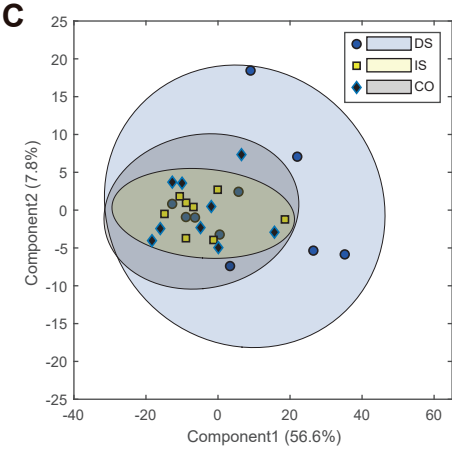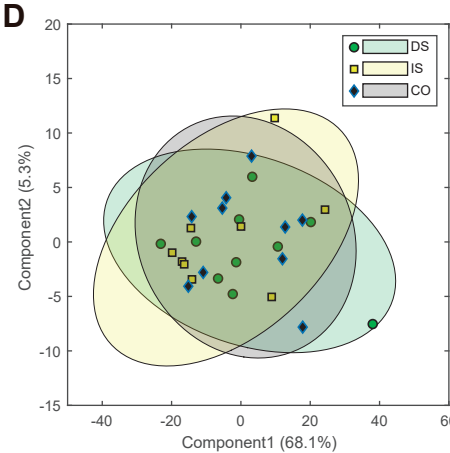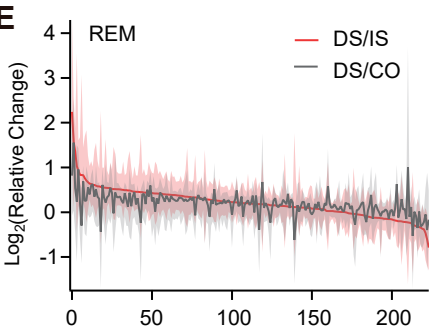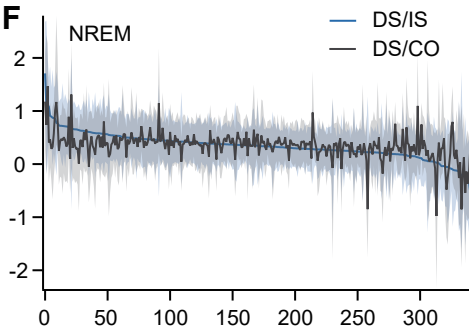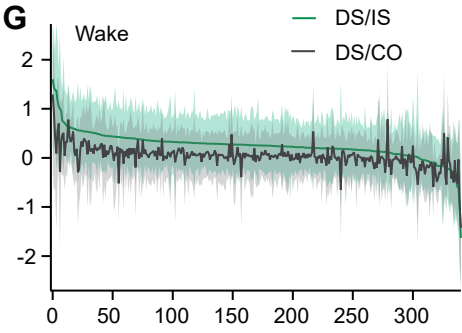

Supplementaly Figure 3

A

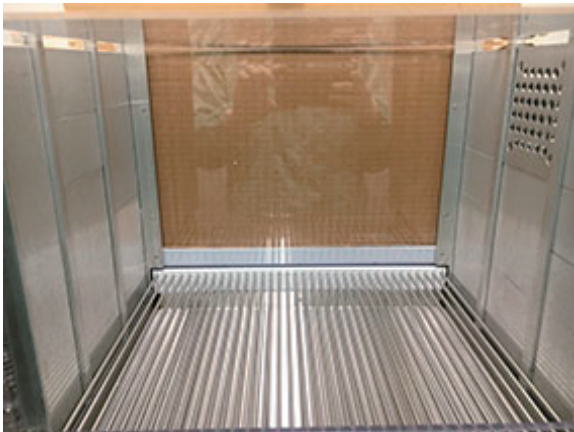

B

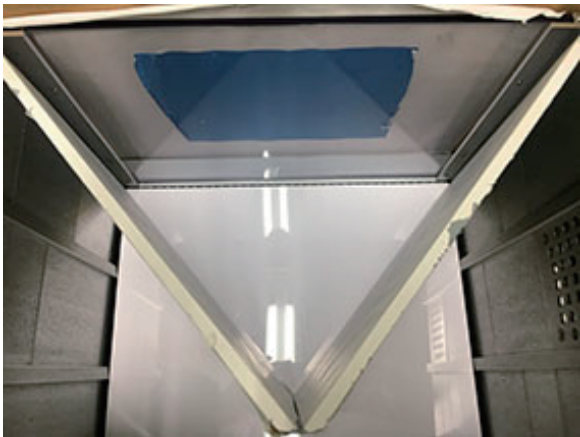

C

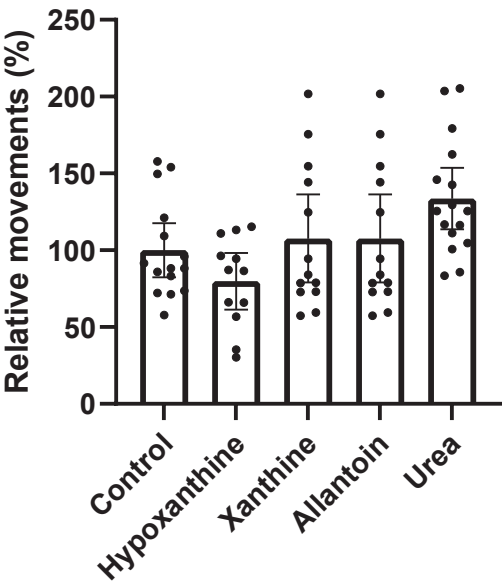

Supplement: Supplementary file 4 — Additional file 4: Fig. S1. Metabolomic changes across sleep–wake states, related to Fig. 1. (A) PCA plots from each group of mice for each sleep–wake state. Each point reflects one mouse, and ellipses represent 95% CIs. (B-C) Fold change of each metabolite between REM vs. Wake groups; red, NREM vs. Wake; blue (B), REM vs. Wake; green (same as in B), REM vs. NREM (C). Fig. S2. PCA analysis found three outliers in the NREM group, related to Fig. 2 . (A-D) PCA plots from each group of mice in each sleep–wake state: REM sleep (A), NREM sleep before (B) and after (C) eliminating outliers (two in the IS group, one in the DS group) that fell outside the 95% CIs ellipses, or wakefulness (D). Each point reflects one mouse. DS-REM group, n = 11 mice; IS-REM, n = 7; CO-REM, n = 9; DS-NREM, n = 10 (after eliminating one outlier); IS-NREM, n = 8 (after eliminating two outliers); CO-NREM, n = 9; DS-Wake, n = 10; IS-Wake, n = 9; CO-Wake, n = 10. Ellipses represent 95% CIs. (E–G) Fold change of each metabolite between DS vs. IS and DS vs CO in each sleep–wake state. Fig. S3. Front view of the fear conditioning context, related to Figs. 2, 4and 5. (A-B) Context A (A) for conditoining and the context test and Context B (B) for the tone test. (C) Relative movements during the pre-shock period. Control, n = 16 mice; allantoin, n = 19; xanthine, n = 13; hypoxanthine, n = 12; urea, n = 16. Kruskal–Wallis test, p < 0.05, Dunn’s multiple comparison tests, p > 0.05 for all comparisons, error bars, 95% CIs. [file 13041_2021_733_MOESM4_ESM.pdf]
